# Phytoremediation Perspectives of Seven Aquatic Macrophytes for Removal of Heavy Metals from Polluted Drains in the Nile Delta of Egypt

**DOI:** 10.3390/biology10060560

**Published:** 2021-06-20

**Authors:** Mohamed Abdelaal, Ibrahim A. Mashaly, Dina S. Srour, Mohammed A. Dakhil, Mohamed Azab El-Liethy, Ali El-Keblawy, Reham F. El-Barougy, Marwa Waseem A. Halmy, Ghada A. El-Sherbeny

**Affiliations:** 1 Department of Botany, Faculty of Science, Mansoura University, Mansoura 35516, Egypt; iamashaly1950@mans.edu.eg (I.A.M.); dodoo.srour@gmail.com (D.S.S.); 2 Botany and Microbiology Department, Faculty of Science, Helwan University, Cairo 11790, Egypt; mohamed_dakhil@science.helwan.edu.eg; 3 Environmental Microbiology Lab., Water Pollution Research Department, National Research Centre, Giza 12622, Egypt; ma.el-liethy@nrc.sci.eg; 4 Department of Applied Biology, Faculty of Science, University of Sharjah, Sharjah 27272, United Arab Emirates; akeblawy@sharjah.ac.ae; 5 Department of Botany and Microbiology, Faculty of Science, Damietta University, New Damietta 34517, Egypt; reham_fekry2012@du.edu.eg; 6 Department of Environmental Sciences, Faculty of Science, Alexandria University, Alexandria 21511, Egypt; marwa.w.halmy@alexu.edu.eg

**Keywords:** heavy metals, bioaccumulation, phytostabilization, phytoextraction, emergent hydrophytes, species diversity

## Abstract

**Simple Summary:**

Some main drains in the Nile Delta of Egypt are subjected to heavy pollution loads and used to irrigate crops and vegetables. Here, we assessed the pollution level and the ability of some wild aquatic macrophytes (*Cyperus alopecuroides*, *Echinochloa stagnina*, *Eichhornia crassipes*, *Ludwigia stolonifera*, *Phragmites australis*, *Ranunculus sceleratus*, and *Typha domingensis*) to accumulate eight heavy metals (Fe, Cu, Zn, Mn, Co, Cd, Ni, and Pb) in three of the polluted drains (Amar, El-Westany, and Omar-Beck). The sediment in the three drains exceeded the worldwide permissible ranges of Cu, Zn, and Pb, but it ranged within safe limits for Mn, Cd, Ni, and Co. *P. australis* accumulated the highest levels of Fe, Co, Cd, and Ni, while *E. crassipes* contained the highest concentrations of Cu, Zn, Mn and Pb. The bioaccumulation factor was > 1 for the investigated heavy metals (except Cu) in all species, except *C. alopecuroides*. Accordingly, these species could be applied for the accumulation and phytostabilization of these metals.

**Abstract:**

The current study addressed the heavy metals accumulation potentials of seven perennial aquatic macrophytes (*Cyperus alopecuroides*, *Echinochloa stagnina*, *Eichhornia crassipes*, *Ludwigia stolonifera*, *Phragmites australis*, *Ranunculus sceleratus* and *Typha domingensis*) and the pollution status of three drains (Amar, El-Westany and Omar-Beck) in the Nile Delta of Egypt. Nine sites at each drain were sampled for sediment and plant analyses. Concentrations of eight metals (Fe, Cu, Zn, Mn, Co, Cd, Ni, and Pb) were determined in the sediment and the aboveground and belowground tissues of the selected macrophytes. Bioaccumulation factor (BF) and translocation factor (TF) were computed for each species. The sediment heavy metals concentrations of the three drains occurred in the following order: El-Westany > Amar > Omar-Beck. The concentrations of sediment heavy metals in the three drains were ordered as follows: Fe (438.45–615.17 mg kg^−1^) > Mn (341.22–481.09 mg kg^−1^) > Zn (245.08–383.19 mg kg^−1^) > Cu (205.41–289.56 mg kg^−1^) > Pb (31.49–97.73 mg kg^−1^) > Cd (13.97–55.99 mg kg^−1^) > Ni (14.36–39.34 mg kg^−1^) > Co (1.25–3.51 mg kg^−1^). The sediment exceeded the worldwide permissible ranges of Cu, Zn and Pb, but ranged within safe limits for Mn, Cd, Ni and Co. *P. australis* accumulated the highest concentrations of Fe, Co, Cd and Ni, while *E. crassipes* contained the highest concentrations of Cu, Zn, Mn, and Pb. Except for *C. alopecuroides* and Cu metal, the studied species had BF values greater than one for the investigated heavy metals. Nevertheless, the TFs of all species (except Cd in *L. stolonifera*) were less than one. Hence, the studied species are appropriate for accumulation, biomonitoring, and phytostabilization of the investigated metals.

## 1. Introduction

During the twenty-first century, major challenges to humanity are associated with water quantity and quality. These challenges will be aggravated in the future due to anthropogenic activities and climate change [1]. In many developing countries, significant population increase, which is associated with increases in industrial and agricultural land use, has led to incredible rises in the discharge of different pollutants into waterways and created adverse effects upon elements of the aquatic environment. Water scarcity is the main problem that will face Egypt shortly, due to excessive population growth and the completion of construction of the Grand Ethiopian Renaissance Dam on the Nile River [2]. Moreover, recent unjustifiable agricultural and human activities have launched numerous polluting substances such as domestic wastewater, chemical fertilizers, organic matter, and herbicides into the Nile River and associated drains [3]. Therefore, it is urgent to overcome the predicted drought problems by managing available water resources, especially remediation and reuse of wastewater [2].

Contamination of freshwater bodies has become a major global concern. High levels of heavy metals from anthropogenic activities, non-biodegradable industrial wastes, sewage, and chemical fertilizers are released into rivers and streams and cause major global health and environmental problems [4,5]. In particular, the risk of heavy metals is attributed to their toxicity, permanence, and bioaccumulation in the environment [6]. If these metals are released into the sediment/soil or water, they cannot be degraded and will reach the food chain through plants and aquatic animals [7,8,9]. Common heavy metals such as cobalt (Co), cadmium (Cd), nickel (Ni), lead (Pb), etc., are toxic at both low and high concentrations in the natural environment. At low levels, specific essential metals (e.g., Fe, Mn, Zn, Cu, etc.) are nutritionally important for a healthful life; nevertheless, they may cause toxicity at very high concentrations, depending on their mobility and solubility [10]. Moreover, poor treatment of wastewater increases wetlands contamination by discharging considerable amounts of heavy metals and nutrients into surface waters, which, in sequence, proliferates algal blooms, reduces O_2_, and reduces wetlands’ productivity [11].

From economic and ecological perspectives, the use of plants as water purifiers/phytoremediators in various aquatic ecosystems is receiving greater interest [9,12,13,14,15,16,17]. Phytoremediation is an eco-friendly, inexpensive, and widely acceptable remediation technology employing plants and associated microorganisms for the treatment of polluted sediment/soil and water and the recovery of soil and water properties [18,19,20,21]. Different approaches are used to achieve phytoremediation, such as phytostabilization, phytoextraction, and rhizofiltration [22]. Phytostabilization is the capability of a plant species to immobilize metal and accumulate it in its belowground tissues. Phytoextraction is the removal of metals and pollutants by belowground roots from the sediment/soil or water [23]. Aquatic macrophytes and wetland plants are perfect tools in phytoremediation approaches due to their ability to accumulate and store high concentrations of metals from the surrounding environments (water or sediment) via their belowground tissues/roots [24] and translocate them to their aboveground tissues (stem or leaf, etc.) without hindering plant growth [25].

In the last few years, three main drains (Amar, El-Westany and Omar-Beck) along the Nile Delta of Egypt have been exposed to exponential and severe pollution by anthropogenic activities. Among others, industrial, agricultural and municipal wastes have contaminated these drains with heavy metals and other solid nondegradable contaminants. Although these three drains have a heavy pollution load, which does not fulfill authorized standards, they are used to irrigate crops and vegetables [26]. No previous studies have addressed the pollution status and heavy metal content of these three drains to our knowledge. In this study, we selected seven perennial aquatic macrophytes (*Cyperus alopecuroides* Rottb., *Echinochloa stagnina* (Retz.) P. Beauv., *Eichhornia crassipes* (C. Mart.) Solms, *Ludwigia stolonifera* (Guill. & Perr.) P.H. Raven, *Phragmites australis* (Cav.) Trin. ex Steud, *Ranunculus sceleratus* L. and *Typha domingensis* Pers.) to assess their phytoremediation potentials. These plants are widely distributed with high biomass, and displayed fast growth within the investigated three drains. They also have demonstrated high abilities to bioaccumulate heavy metals in several drains in Egypt and the world [9,14,15,24,27,28]. Therefore, we postulated that the selected seven plant species and their tissues (aboveground and belowground) have the ability to hyperaccumulate and translocate eight heavy metals—iron (Fe), copper (Cu), zinc (Zn), manganese (Mn), cadmium (Cd), cobalt (Co), nickel (Ni), and lead (Pb)—from the contaminated drains at different degrees. Hence, the main objectives of the present study were to 1) estimate the concentrations of heavy metals in the sediment of the three drains to address the most polluted one, and 2) evaluate the phytoremediation potentials of the selected plant species for the investigated heavy metals.

## 2. Materials and Methods

### 2.1. Study Area

**Figure 1:**
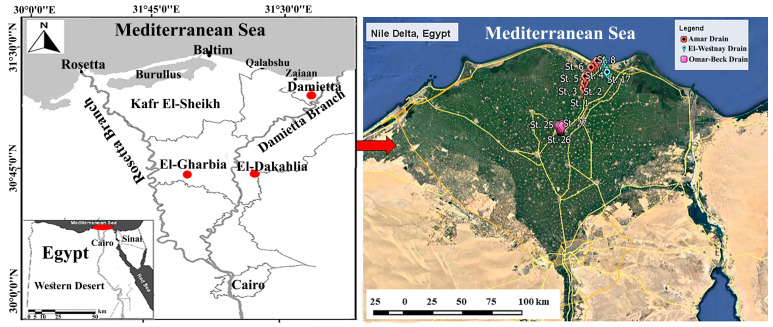
Map of the Nile Delta of Egypt showing the sampling sites (St. 1–St. 27) in the three drains (Amar, El-Westany, and Omar-Beck). Map source: Google Earth Pro v. 7.3.3.7886, accessed on 25 August 2020.

The current study was carried on three drains: Amar, El-Westany, and Omar-Beck (Figure 1). These drains lie within the borders of three governorates, Damietta, El-Dakahlia, and El-Gharbia, in the Nile Delta of Egypt. The first two drains were constructed to transfer agricultural wastes from the Nile Delta and discharge them into the Mediterranean Sea. The third (Omar-Beck) directly discharges into the Nile River. The length and width of the Amar Drain are about 20 km and 45 m, respectively, and it completely extends within El-Dakahlia Governorate, beginning in Bilqas City and emptying into the Mediterranean Sea in Gamasa City; it receives pollution and discharges from fertilizer factories, agricultural drainages, industrial wastes, and sewage effluents. El-Westany Drain extends for ca. 17 km with a width of ca. 55 m and receives wastes from cheese and furniture/painting factories, agricultural drainage, and municipal wastes. Omar-Beck Drain extends for about 12 km in length, is ca. 40 m in width, and receives wastes from agricultural drainages, textile factories, and linen maceration. The main source of water in the study area is the Nile River. The pH of water samples was 7.90 for Amar Drain, 7.97 for El-Westany Drain, and 7.79 for Omar-Beck Drain. In addition, the electric conductivity (EC) ranged between 1.49 dS/m for Amar, 2.62 dS/m for El-Westany, and 1.18 dS/m for Omar-Beck [29].

### 2.2. Sampling Design and Processing

For each drain, nine sites (three each per upstream, midstream, and downstream sectors) were sampled during spring 2020 (Figure 1). Each site was at least one km away from the neighboring site. At each site, a composite sample of sediment soil (*n* = 3) at a profile of 0–30 cm was collected. At the same time, composite samples of seven perennial plant species (*Cyperus alopecuroides*, *Echinochloa stagnina*, *Phragmites australis*, *Ranunculus sceleratus* and *Typha domingensis*, *Eichhornia crassipes* and *Ludwigia stolonifera*) were sampled from each drain. To avoid age bias among selected plants, healthy and fully mature individuals with well-developed aboveground and belowground organs were collected. The choice of sites within the three drains was based on pollution degree and the occurrence of the studied species. In the current study, all of the selected plant species were naturally growing in at least three sites along the studied drains. The plant species were identified according to Boulos [30].

### 2.3. Sediment Analysis

The sediment samples were collected, then air-dried, ground, and sieved via a 2 mm mesh. A 2 g dry sample was digested with 20 mL of HNO_3_:HClO_4_ (2:1 *v*/*v*), then heated in a water bath at 100 °C until clear (~5 mL extract), brought to a constant volume with de-ionized water. The concentrations of heavy metals (Fe, Cu, Zn, Mn, Cd, Co, Ni, and Pb) were estimated using an atomic absorption spectrophotometer (Shimadzu AA-6200 model, Shimadzu Co., Kyoto, Japan) [31]. To confirm accuracy during analysis, a standard stock solution with a known concentration of each studied metal was considered for comparison according to the certified reference values.

### 2.4. Plant Analysis

Healthy plants were sampled in plastic bags. In the lab, the samples were washed with distilled water to remove debris and dust, separated into aboveground and belowground tissues, dried at 60 °C until full dryness (constant weight), and ground into a powder. Two grams each of aboveground and belowground organs were digested and heated in 20 mL of HNO_3_:HClO_4_:H_2_SO_4_ (1:1:5 *v*/*v*/*v*) until a transparent colour is developed. The mixture was filtered, and the filtrate was completed to a specific volume with de-ionized water [9,32]. The concentrations of heavy metals (Fe, Cu, Zn, Mn, Cd, Co, Ni, and Pb) were estimated using an atomic absorption spectrophotometer (Shimadzu AA-6200 model, Shimadzu Co., Japan).

To satisfy quality assurance protocol during sediment and plant analysis, the following settings were monitored: operational and instrumental settings were performed according to the manufactures’ specifications; de-ionized water, blank samples for instrument readings correction, and cleaned glassware were used; triplicate samples and analytical-grade chemicals were used during digestion and analysis; and finally, standard solutions with known concentrations of different studied metals were used for calibration.

### 2.5. Phytoremediation Potentials of the Selected Aquatic Macrophytes

The proficiency of the aboveground and belowground organs of the selected macrophytes for heavy metals accumulation from sediment was calculated using two factors: bioaccumulation factor (BF) and translocation factor (TF). BF estimates the capability of plant organs to uptake the heavy metal from the sediment; TF is the translocation of heavy metal from the belowground organs to the aboveground organs. Here, BF and TF were calculated according to the following equations:

BF = C_Belowground_/C_Sediment_,

TF = C_Aboveground_/C_Belowground_,

where C_Aboveground_ and C_Belowground_ indicate the heavy metal concentrations (mg kg^−1^) in the aboveground and belowground tissues of the selected plant species, respectively, and C_Sediment_ is the heavy metal concentration in the corresponding sediment [27,33,34,35].

### 2.6. Data Analysis

To offer an indication of the sediment heavy metal content in the three drains, box plots including the maximum, minimum, and mean values ± standard errors were used. Before ANOVA, the data were tested for homogeneity of variance. A Kruskal–Wallis one-way ANOVA with a pairwise comparison was applied to test the significant differences among sediment variables (*p* ≤ 0.05) in the three drains, and the heavy metals concentration and phytoremediation factors (BF and TF) among the selected plants. In addition, a three-way ANOVA was applied to test the variation significance among drains, plant species, and tissues (aboveground and belowground) and their interactions on the heavy metal concentration. Finally, to address the relationships between concentrations of the investigated heavy metals within the different plant species/tissues, Pearson correlation coefficients were calculated using a ‘qgraph’ R package with Bonferroni’s correction at *p* ≤ 0.05. All statistical analyses were carried out by using XLSTAT (v. 2016) and R-language [36].

## 3. Results

### 3.1. Sediment Heavy Metals in the Investigated Three Drains

**Figure 2:**
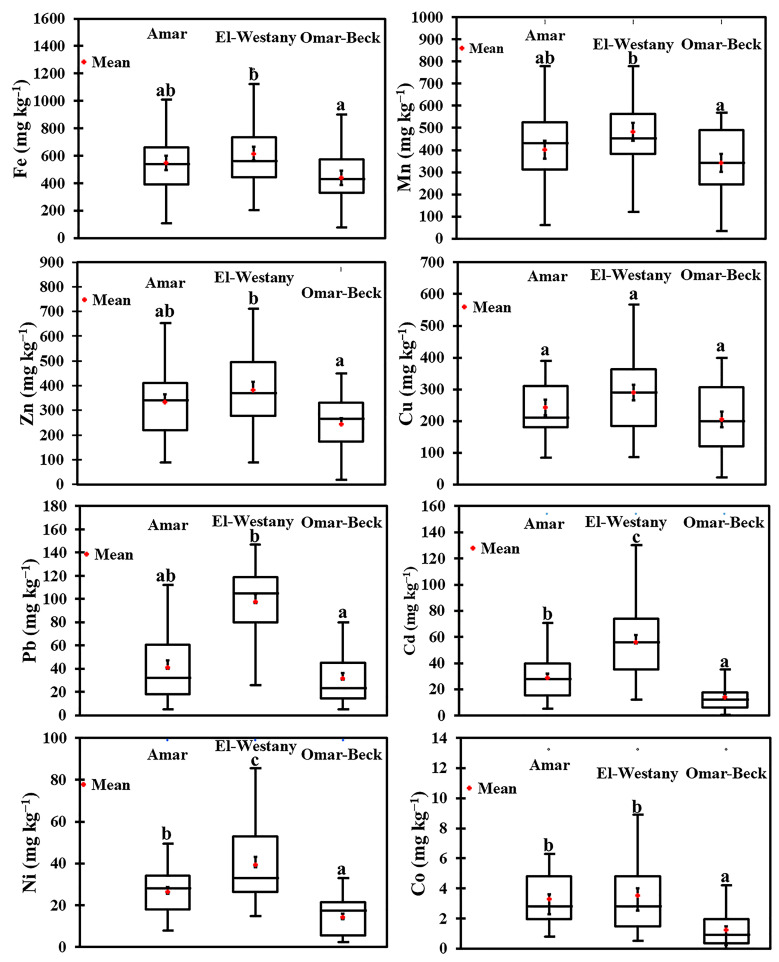
Box plots show the mean values (+red) and standard errors (internal vertical bars) of the measured sediment heavy metals of the three drains (Amar, El-Westany, and Omar-Beck) in the Nile Delta of Egypt. External upper and lower vertical bars display the maximum and minimum values of heavy metals, respectively. Different letters indicate statistically significant differences at *p* ≤ 0.05 according to the Kruskal–Wallis test.

Except for copper (Cu), the concentrations of sediment heavy metals significantly differed among the three studied drains (*p* ≤ 0.05) (Figure 2). Notably, the sediment heavy metals concentrations occurred in the following order: El-Westany > Amar > Omar-Beck. In both Amar and El-Westany Drains, the sediment concentration of heavy metals was in the order of Fe > Mn > Zn > Cu > Pb > Cd > Ni > Co, while for Omar-Beck Drain, the order was similar but with higher concentrations of Ni than Cd. The highest mean concentrations of sediment Fe, Cu, Zn, Mn, Co, Cd, Ni, and Pb (615.17, 289.56, 383.19, 481.09, 3.51, 55.99, 39.34, and 97.75 mg kg^−1^, respectively) were recorded in El-Westany Drain, while the lowest mean concentrations of these metals (438.45, 205.41, 245.08, 341.22, 1.25, 13.97, 14.36 and 31.49 mg kg^−1^, respectively) were recorded in Omar-Beck Drain (Figure 2).

### 3.2. Plant Heavy Metals

The heavy metal concentrations in the different tissues (aboveground and belowground) of the seven studied species are displayed in Table 1. The ANOVA test indicated significant variations in the heavy metals among the different species and tissue location (*p* ≤ 0.05). Except for Cd in *L. stolonifera*, the studied species accumulated higher concentrations of all heavy metals in their belowground tissues than in the aboveground tissues. Among the studied species, *P. australis* stored higher concentrations of Fe, Co, Cd, and Ni in its tissues than the other plant species, while *E. crassipes* contained the highest concentrations of Cu, Zn, Mn, and Pb. The highest concentrations of Fe, Co, Cd and Ni (2043.83, 57.69, 137.30, and 149.40 mg kg^−1^, respectively) were noted in the belowground tissues of *P. australis*, while the lowest concentrations of Fe and Cd (71.96 and 4.66 mg kg^−1^, respectively) were recorded in *R. sceleratus* aboveground tissues, and the lowest concentrations of Co and Ni (3.07 and 4.54 mg kg^−1^, respectively) were found in the aboveground tissues of *C. alopecuroides*. The belowground tissues of *E. crassipes* had the highest concentrations of Cu, Zn, Mn, and Pb (147.70, 553.27, 1783.20, and 344.00 mg kg^−1^, respectively), while the belowground tissues of *C. alopecuroides* attained the lowest concentration of Cu (6.34 mg kg^−1^), and the belowground tissues of *R. sceleratus* had the lowest concentrations of Zn, Mn, and Pb (18.30, 18.00, and 19.50 mg kg^−1^, respectively). Approximately, for aboveground and belowground tissues, the heavy metal concentration occurred in the descending order: Fe > Mn > Zn > Pb > Cu > Ni > Cd > Co.

### 3.3. Interactive Effects of Drains, Plant Species, and Tissues on Sediment’s Heavy Metals

The three-way ANOVA results displayed significant effects of drain, plant species, and tissue and their interactions on most of the investigated heavy metals (Table 2). Fe concentration was influenced by all factors except tissue and the interaction between drain and tissue. Cu concentration was significantly affected by all factors except drain and drain × tissue. Zn, Mn, Cd, and Pb concentrations were significantly shaped by all factors and all of their interactions. Co concentration was variable, depending on species, the interaction of drain with either species or species × tissue, and species × tissue interactions. Ni level depended significantly on species, drain × species, species × tissue, and drain × species × tissue interactions.

**Figure 3:**
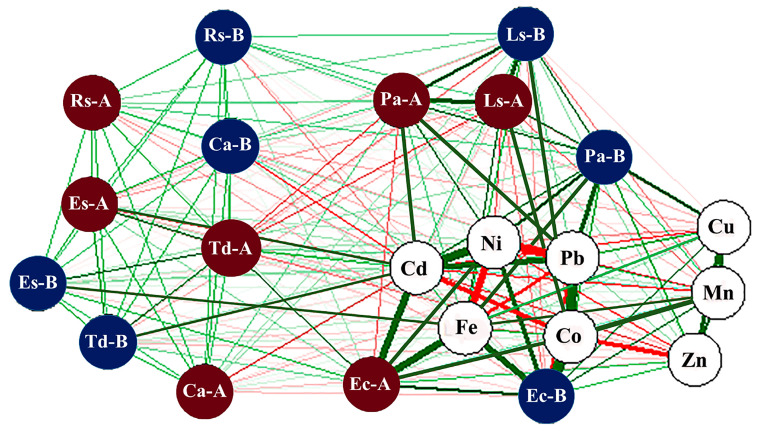
Pearson correlation network of heavy metals (Fe, Cu, Zn, Mn, Cd, Co, Ni, and Pb) in the aboveground (dark red circles) and belowground (blue circles) tissues of seven plant tissues. Positive correlations are indicated in green gradient color, while negative correlations are in red gradient. Ec-A and Ec-B: *Eichhornia crassipes* aboveground and belowground tissues, respectively; Es-A and Es-B: *Echinochloa stagnina* aboveground and belowground tissues, respectively; Ca-A and Ca-B: *Cyperus alopecuroides* aboveground and belowground tissues, respectively; Ls-A and Ls-B: *Ludwigia stolonifera* aboveground and belowground tissues, respectively; Pa-A and Pa-B: *Phragmites australis* aboveground and belowground tissues, respectively; Rs-A and Rs-B: *Ranunculus sceleratus* aboveground and belowground tissues, respectively; Td-A and Td-B: *Typha domingensis* aboveground and belowground tissues, respectively.

The results of the Pearson correlation network of the eight heavy metals’ concentrations within the tissues (aboveground and belowground) of the seven plants are displayed in Figure 3. *P. australis* belowground tissue was significantly positively correlated with Fe, Cu, Ni, Cd and Pb; *P. australis* aboveground tissue was significantly positively proportional to Cd, Pb and Ni. Aboveground tissue of *E. crassipes* was positively correlated with Fe and Cd, Ni and Co. Concentrations of Fe, Cu, Zn, Mn, Ni, and Co were positively correlated with the belowground tissue of *E. crassipes*. The tissues of *T. domingensis* were positively linked with Cd; *L. stolonifera* tissues with Ni, Co, and Cd; and *E. stagnina* tissues with Fe and Cd. The tissues of *C. alopecuroides* were negatively correlated with Cd. Positive correlations were found between Fe and Mn; Fe and Cu; Cu and Mn; Mn and Zn; Ni and Cd; Cd and Pb; Pb and Co. However, negative correlations were observed between Fe, Ni, and Pb; Cd and Co; Pb and Ni, Cu; and Co and Zn.

### 3.4. Heavy Metals Phytoremediation Assessment for Plant Species

The bioaccumulation factor (BF) and translocation factor (TF) of the heavy metals are presented in Table 3. Excluding *C. alopecuroides* and Cu, the studied species had BFs values greater than one for the other investigated heavy metals. For Fe, four species (*P. australis*, *E. crassipes*, *E. stagnina* and *T. domingensis*) had BFs > 1 (3.83, 3.42, 2.43, and 1.52, respectively). Among the tested species, only *E. crassipes* had a high BF for Zn (1.72). For Mn, the highest BF values of > 1 were recorded in four species in the following order: *E. crassipes* (4.37), *P. australis* (3.99), *E. stagnina* (2.29) and *T. domingensis* (2.28). Five species showed BF values of > 1 for Co; these species include *P. australis* (5.49), *T. domingensis* (3.29), *E. crassipes* (2.89), *L. stolonifera* (1.21) and *R. sceleratus* (1.02). Regarding Cd, five species had high BF values > 1; these species are *P. australis* (5.78), *L. stolonifera* (2.02), *T. domingensis* (1.83), *E. crassipes* (1.67) and *E. stagnina* (1.05). *P. australis*, *E. crassipes*, *T. domingensis*, *L. stolonifera*, *E. stagnina* and *R. sceleratus* showed the highest BFs value > 1 for Ni (BF: 5.59, 4.25, 4.02, 3.52, 3.18, and 2.42, respectively). For Pb, *E. crassipes* had the highest BF value (6.06), followed by *L. stolonifera* (3.69), *P. australis* (2.87), *E. stagnina* (2.59) and *T. domingensis* (1.13). The lowest BF values < 1 for Fe, Cu, Zn, Mn, Co and Ni were recorded in *C. alopecuroides* (BF: 0.25, 0.07, 0.16, 0.07, 0.62, and 0.37, respectively), while *R. sceleratus* had the lowest BFs for Cd and Pb (0.22 and 0.62, respectively). On the other hand, except for the TF of Cd in *L. stolonifera* (1.59), the TFs of the other species for all heavy metals did not exceed one. However, *C. alopecuroides* had the highest TF for Fe (0.89), *T. domingensis* for both Cu and Zn (0.60 and 0.83, respectively), *E. stagnina* for Mn (0.84), *L. stolonifera* for Co, Cd, and Pb (0.79, 1.59 and 0.99, respectively) and *E. crassipes* for Ni (0.81).

## 4. Discussion

According to the sediment heavy metals concentrations, El-Westany Drain is the most polluted studied drain in the Nile Delta of Egypt, followed by Amar Drain and Omar-Beck Drain. The concentration and bioavailability of heavy metals in sediment depend on natural origin, discharge rate, pH, organic matter content, soil texture, competitive ions, root exudates, wastes type, etc. [5,27,37,38]. In addition to the earth’s crust, which is the primary source of background metals, human activities significantly contribute to increasing these metals in the sediment [39]. Consequently, sediment pollution with heavy metals is one of the main issues posing environment and human health risks.

In the current study, the high degree of pollution in El-Westany Drain, as compared with the other two drains (Amar and Omar-Beck), may be attributed to the huge amounts and different sources of sewages and agricultural drainages which directly discharge into its stream without any treatment stations [26]. In the present study, the concentrations of sediment heavy metals were ordered as Fe > Mn > Zn > Cu > Pb > Cd > Ni > Co. These results agree with Fawzy et al. [24] and El-Amier et al. [40], who found a similar pattern of heavy metals distribution in different canals and drains along the Nile River of Egypt. Fe and Mn were the most abundant metals in the sediment in the three drains among the investigated metals. This finding is in accordance with the fact that Fe and Mn are the most common and associated metals in the earth’s crust and usually exist in oxide and hydroxide forms [41]. Conversely, the high concentrations of sediment Fe and Mn may be attributed to anthropogenic activities, runoff of sewage, fertilizers, etc. [42,43]. Furthermore, the observable elevated concentrations of sediment Zn and Cu are linked with soluble carbonates, oxides, and organic matter content [44]. As aforementioned, the degree of human impacts on watercourses substantially affects heavy metals concentration and consequently the degree of pollution. In this context, sewage sludge, synthetized fertilizers, paints, dyes, electroplating, steel industries, batteries, pipes, pesticides, soaps, antimicrobial agents, fuel combustion, etc. likely greatly contribute to the raised levels of the studied heavy metals in the studied drains of the Nile River in Egypt [19,24,26,40,45]. On the other hand, the low concentrations of Cd, Ni, and Co might suggest a probable sole natural origin.

The mean concentrations of Cu, Zn, and Pb in the sediment samples collected from the three drains exceeded the worldwide safe permissible ranges of Cu (38.9 mg kg^−1^), Zn (70 mg kg^−1^), and Pb (27 mg kg^−1^) [46]. In contrast, the concentrations of sediment Mn, Cd, Ni, and Co ranged within the safe limits (488 mg kg^−1^ of Mn, 41 mg kg^−1^ of Cd, 29 mg kg^−1^ of Ni, and 11.3 mg kg^−1^ of Co). The investigated drains contained a higher level of Cd, but lower levels of Ni and Pb, than other drains of the Nile Delta, such as the Kitchener drain that had 4.10 mg kg^−1^ of Cd, 68.7 mg kg^−1^ of Ni, and 83.4 mg kg^−1^ of Pb [9]. Furthermore, the data of this study indicated that the mean values of the estimated heavy metals were lower than that reported by [40] for three drains (Elhoks, El-Shakhlouba and Drain 7), [45] for the Kitchener Drain, and [47] for contaminated wetlands in Egypt. In addition, Eid et al. [48] recorded 11.9–12.6 mg kg^−1^ of Ni and 12.7–13.7 mg kg^−1^ of Pb in the sediment of Burullus Lake of Egypt, which tended to be lower than that estimated in this study.

The ability of aquatic macrophytes to hyperaccumulate and translocate heavy metals varies depending on the study site, species, tissues, pH, and redox potential [9,49,50]. Our results indicate that *P. australis* stored the highest concentrations of Fe, Co, Cd, and Ni, while *E. crassipes* collected the highest levels of Cu, Zn, Mn, and Pb. Nevertheless, the other tested species accumulated considerable concentrations of the investigated heavy metals. Our results differ from those reported in other studies on the same plants in relevant canals and drains in Egypt or the world. This might be explained by differences in the pollution degree, sampling time, extraction and analytical methods, and physical and chemical properties of the watercourse [9,50,51]. For example, Cd, Ni, and Pb concentrations in *P. australis* and *E. crassipes* were higher than those recorded for the same species in the Kitchener Drain in the Nile Delta [9]. By contrast, Eid et al. [9] showed higher concentrations of Cd, Ni, and Pb for *E. stagnina* and *L. stolonifera* than the same species in our study. Galal et al. [28] reported higher concentrations of Fe, Cu, Zn, and Mn but lower concentrations of Cd, Ni, and Pb for *L. stolonifera* roots in the Greater Cairo watercourses as compared with this study. Moreover, the shoots and roots of *C. alopecuroides* showed higher concentrations of the investigated metals in contaminated wetlands than in our study [47]. Furthermore, Batool et al. [52] proposed a negative correlation between Ni and Cd concentrations and the growth and biomass of *C. alopecuroides* in polluted wetlands. The Cu, Mn, Cd, Ni, and Pb concentrations in the tissues of both *P. australis* and *T. domingensis* were higher than those reported by [53] in a polluted estuary in Italy and [19,54] for *P. australis* and *T. domingensis* organs in Burullus Lake, north Egypt. In addition, the Fe and Mn levels in *E. crassipes* were higher, and the Cu, Zn, Co, Cd, Ni, and Pb concentrations were lower, than those documented by [27] in the Nile Delta and [55] in contaminated coastal water in Nigeria for the same species. In the current study, the tissues of *R. sceleratus* showed higher concentrations of Cd, Ni, and Pb but lower Mn than the same species studied in [14].

Regarding the ordinarily safe and phytotoxic levels of heavy metals, the studied species had high concentrations of Cd and Pb that exceeded the permissible safe levels of Cd (5–30 mg kg^−1^) and Pb (30–300 mg kg^−1^) in wild plants and crops [46,47]. Except for *C. alopecuroides* tissues, the studied species accumulated a high level of Cu, which ranged within the phytotoxic level (20–100 mg kg^−1^). In addition, the Fe and Zn concentrations in *P. austarlis*, *E. stagnina* and *E. crassipes* tissues, the Mn concentration in *P. australis* and *E. crassipes* tissues, the Co and Ni concentrations in *P. austarlis*, *T. domingensis* and *E. crassipes* tissues were higher than the safe levels in plants (phytotoxic levels of Fe: >500 mg kg^−1^, Zn: 100–400 mg kg^−1^, Mn: >400 mg kg^−1^, Co: 15–50 mg kg^−1^ and Ni: 10–100 mg kg^−1^) [46]. These results coincided with those reported in the same species in previous studies [5,24,27,40,47]. The notable high concentrations of most investigated heavy metals in the studied species may be in response to the high concentration and bioavailability of these metals in the sediment in the three drains. Moreover, the plant species that contained a high concentration of a metal exceeding or within the phytotoxic range might have the capacity for hyperaccumulation of that metal [14]. One of the most realistic and essential features of hyperaccumulators is that the species should tolerate very high concentrations of metals (10,000 mg kg^−1^ for Zn and Mn; >1000 mg kg^−1^ for Cu, Co, Ni and Pb; and 100 mg kg^−1^ for Cd) [9,12,56]. Accordingly, the studied species are regarded as potential accumulators rather than hyperaccumulators for the investigated metals.

The belowground tissues of all studied species accumulated higher concentrations of metals, except for Cd in *L. stolonifera*, than the above-ground tissues. This finding agrees with those previously reported for the same species [9,12,15,16,19,27,28,47]. The hyperaccumulation of heavy metals in the belowground roots of aquatic macrophytes has been considered a protection mechanism against metal toxicity through two pathways: compartmentalization (sequestration of high concentrations of heavy metals within the roots) and exclusion [9,12,57,58]. Furthermore, Sawidis et al. [59] attributed the high concentrations of metals in the belowground roots to the adsorption of these metals on the roots, and the large surface area and thick parenchyma cells of roots. Other studies [22,24,27,60] attribute the greater accumulation of heavy metals in roots variously to the fact that roots are the first plant organs in direct contact and uptake with heavy metals, therefore retaining more metals, or to the presence of phytochelatins that help in metals sequestration, or to sulfhydryl groups. Likewise, [9,16,61] observed that emergent plants, such as *P. australis* and *T. domingensis*, can immobilize toxic metals, in particular, Cd and Pb, and consequently store them in their roots. By contrast, in *L. stolonifera*, the high accumulation of Cd in the aboveground tissues may be attributed to its compartmentalization in the leaf vacuoles [9,50].

The capability of the studied species to accumulate high levels of Fe, Cu, Zn, Mn, Co, Cd, Ni, and Pb in their belowground tissues makes them potential candidates for phytoextraction, phytostabilization, and sequestration of these metals. Two phytoremediation factors, bioaccumulation factor (BF) and translocation factor (TF), are used to estimate the phytoremediation potentials of plants [14,27,62]. According to [13,14,35], species with BFs value > 1 and TFs < 1 might be useful for phytostabilization purposes, while species with BFs = TFs > 1 might be suitable for phytoextraction purpose. The studied species, except *C. alopecuroides*, can be ranked according to BFs for the investigated heavy metals as follows: *P. australis* > *E. crassipes* > *E. stagnina* > *T. domingensis* for Fe; *E. crassipes* for Zn; *E. crassipes* > *P. australis* > *E. stagnina* > *T. domingensis* for Mn; *P. australis* > *T. domingensis* > *E. crassipes* > *L. stolonifera* > *R. sceleratus* for Co; *P. australis* > *L. stolonifera* > *T. domingensis* > *E. crassipes* > *E. stagnina* for Cd; *P. australis* > *E. crassipes* > *T. domingensis* > *L. stolonifera* > *E. stagnina* > *R. sceleratus* for Ni; *E. crassipes* > *L. stolonifera* > *P. australis* > *E. stagnina* > *T. domingensis* for Pb. Nevertheless, the TFs of the heavy metals for the investigated plant species were less than one. Hence, these species are appropriate for phytostabilization of these heavy metals, except *L. stolonifera* for Cd, which is seemingly appropriate for phytoextraction of Cd. These results are in harmony with the findings of several studies on aquatic plant species (e.g., [9,14,19,22,27,63,64]). Moreover, on the basis of BF data, most of the studied species may be regarded as hyperaccumulators for Fe, Zn, Mn, Co, Cd, Ni and Pb. Similar results were reported for *P. australis* [9,13,16,24,48,54], *E. crassipes* [9,24,27,63,65,66], *T. domingensis* [5,24], *L. stolonifera* [9,28,67], *R. sceleratus* [14], and *E. stagnina* [9]. Regarding *C. alopecuroides*, the study of [47] reported similar findings, except for Pb, where they displayed the ability of *C. alopecuroides* for Pb phytoextraction. The TFs value below one indicated that the studied species exhibited a lower rate of metals translocation and distribution from the belowground tissues to their aboveground tissues. Therefore, the studied species accumulated heavy metals in their belowground tissues and did not efficiently translocate metals from the belowground roots to the other plant organs. Hence, the studied species might be considered as metals excluders and for phytostabilization of sediment polluted with metals [68,69]. These results were in harmony with several previous studies [9,14,19,53,70]. The main reason for the limited translocation of the investigated metals is the powerful sequestration of these metals in the cortical tissues of roots, which is regarded as a tolerance tool of aquatic macrophytes [53,58,70,71]. However, the essential metals (e.g., Fe, Cu, Zn, Mn) for metabolic activities of plants are frequently differentially allocated to the aboveground organs [70]. Therefore, the mobility/translocation of these metals was significantly varied among the studied species and tissues.

In macrophytes, heavy metals are absorbed from sediment through belowground organs and translocated to the aboveground tissues [72]. Therefore, metal accumulation in plant tissues is controlled by metal availability in the sediment, species, organs uptake, and translocation process [70,73]. The current study displayed significant positive correlations between heavy metal concentrations in the plant species/tissues and the sediment. For example, significant positive correlations were noticed between *P. australis* tissues (aboveground and belowground) and Fe, Cu, Ni, Cd, and Pb; *E. crassipes* tissues and Fe, Zn, Co, Ni, and Cd; *T. domingensis* and Cd; *L. stolonifera* and Ni, Co and Pb; *E. stagnina* and Fe and Cd. These correlations endorsed that these species could be used for biomonitoring and as bioindicators for these metals [9,19]. On the other hand, the strong positive correlations of Fe with Mn and Cu and Mn with Cu, Zn, and Co suggest similar sources for these metals, whether natural or anthropogenic, as well as a translocation or deposition process. A similar pattern of linear increase in the investigated heavy metals’ uptake was previously reported for the same species [9,24,40]. Moreover, [54] reported that concentrations of heavy metals in the environment are positively correlated with their concentrations in various plant organs. On the other hand, the presence of negative correlations between either different metals in the studied plants (e.g., Fe with Ni and Pb; Mn with Cd and Pb; Cd with Co; Zn with Co, etc.) or between species tissues (*P. australis* aboveground tissues with *E. crassipes* aboveground and *T. domingensis* aboveground; *L. stolonifera* tissues with *T. domingensis* aboveground tissues) might support the antagonistic relations hypothesis.

A good candidate plant for phytoremediation and metal accumulation should satisfy the following criteria: potential for rapid growth, well-developed root system, easily harvested, heavily accumulate heavy metals, and high tolerance and wide ecological amplitude [74]. The studied species are characterized by fast growth in the current study and greatly developed underground/underwater tissues. Consequently, all of these features support the studied species as potential phytoremediators/good accumulators for the investigated heavy metals. Finally, to keep phytoremediation proficiency and overcome reaching the lethal level of heavy metals, periodic harvesting of the remediated plant tissues should take place [9,27]. Subsequently, the harvested tissues could be burned into ash and packaged in a protected place for possible metal recovery, if required.

## 5. Conclusions

Concerning sediment heavy metals, El-Westany is among the most polluted drains in the Nile Delta of Egypt, followed by Amar and Omar-Beck Drains. For the three drains, the sediment exceeded the worldwide permissible ranges of Cu, Zn, and Pb, but it ranged within safe limits for Mn, Cd, Ni, and Co. The selected plants accumulated higher concentrations of investigated metals in their belowground tissues than aboveground tissues. *P. australis* accumulated the highest concentrations of Fe, Co, Cd, and Ni while *E. crassipes* attained the greatest concentrations of Cu, Zn, Mn, and Pb. Except for *C. alopecuroides* and Cu metal, the studied species had bioaccumulation factors (BFs) greater than one for the investigated heavy metals, while the translocation factors (TF) of all species (except Cd in *L. stolonifera*) were less than one. According to BFs, the studied species can be ranked as follows: *P. australis* > *E. crassipes* > *E. stagnina* > *T. domingensis* for Fe; *E. crassipes* for Zn; *E. crassipes* > *P. australis* > *E. stagnina* > *T. domingensis* for Mn; *P. australis* > *T. domingensis* > *E. crassipes* > *L. stolonifera* > *R. sceleratus* for Co; *P. australis* > *L. stolonifera* > *T. domingensis* > *E. crassipes* > *E. stagnina* for Cd; *P. australis* > *E. crassipes* > *T. domingensis* > *L. stolonifera* > *E. stagnina* > *R. sceleratus* for Ni; *E. crassipes* > *L. stolonifera* > *P. australis* > *E. stagnina* > *T. domingensis* for Pb. Therefore, the studied species are appropriate for accumulation, biomonitoring, and phytostabilization of the investigated metals. Moreover, the present study displayed positive correlations between heavy metals in the plant species/tissues (*P. australis* with Fe, Cu, Ni, Cd and Pb; *E. crassipes* with Fe, Zn, Co, Ni and Cd; *T. domingensis* with Cd; *L. stolonifera* with Ni, Co and Pb; and *E. stagnina* with Fe and Cd). Such proportional correlations with their high BFs values endorse the ability of these species to accumulate and biomonitor these metals from the polluted drains. The presence of different species (bioaccumulator diversity) may enhance the on-site phytoremediation potential more than a single species, which needs further experimental work. The outcomes of the current study indicate that it is mandatory for the government and relevant authorities to immediately support the following actions: construction of a water treatment plant on El-Westany Drain, outlining a new end/path for Amar Drain to prevent its discharge into the Nile water, and finally managing industrial and municipal sewages.

## Figures and Tables

**Table 1:** Mean values of heavy metals concentration (mg kg^−1^) in the aboveground (AG) and belowground (BG) tissues of the study plant species collected from the three drains in the Nile Delta of Egypt.

Heavy Metal	Plant Species													
	C.a.		E.s.		P.a.		R.s.		T.d.		E.c.		L.s.	
	AG	BG	AG	BG	AG	BG	AG	BG	AG	BG	AG	BG	AG	BG
Fe	118.27 ^ab^	133.53 ^a^	1031.67 ^ab^	1298.23 ^ab^	1465.60 ^b^	2043.83 ^b^	71.96 ^a^	169.28 ^ab^	529.87 ^ab^	813.50 ^ab^	794.30 ^ab^	1826.83 ^ab^	120.49 ^a^	140.18 ^a^
Cu	6.34 ^a^	18.40 ^a^	36.90 ^ab^	66.67 ^ab^	24.18 ^ab^	83.00 ^ab^	23.69 ^ab^	67.73 ^ab^	35.90 ^ab^	59.50 ^ab^	78.47 ^b^	147.70 ^b^	12.55 ^ab^	36.16 ^ab^
Zn	29.73 ^a^	51.11 ^a^	189.33 ^ab^	237.40 ^ab^	86.40 ^ab^	178.77 ^ab^	18.30 ^a^	119.07 ^ab^	79.33 ^ab^	95.37 ^ab^	319.23 ^b^	553.27 ^b^	61.44 ^ab^	138.80 ^ab^
Mn	16.07 ^a^	30.04 ^a^	782.13 ^ab^	933.43 ^ab^	1126.67 ^ab^	1630.00 ^b^	18.00 ^ab^	50.58 ^ab^	591.07 ^ab^	930.68 ^ab^	1163.93 ^b^	1783.20 ^b^	94.48 ^ab^	153.03 ^ab^
Co	3.07 ^a^	6.50 ^a^	6.63 ^ab^	10.27 ^ab^	10.09 ^ab^	57.69 ^b^	5.71 ^ab^	10.79 ^ab^	13.00 ^ab^	34.50 ^ab^	21.17 ^b^	30.33 ^ab^	10.07 ^ab^	12.75 ^ab^
Cd	5.31 ^a^	9.57 ^ab^	13.27 ^ab^	24.93 ^ab^	36.20 ^ab^	137.30 ^b^	4.66 ^a^	5.23 ^a^	21.83 ^ab^	43.40 ^ab^	31.17 ^ab^	39.67 ^ab^	76.20 ^b^	47.97 ^ab^
Ni	4.54 ^a^	9.80 ^a^	26.35 ^ab^	85.00 ^ab^	28.50 ^ab^	149.40 ^b^	16.46 ^ab^	64.72 ^ab^	48.20 ^ab^	107.53 ^ab^	91.40 ^b^	113.50 ^ab^	69.00 ^ab^	94.13 ^ab^
Pb	28.50 ^ab^	36.87 ^a^	50.33 ^ab^	146.80 ^ab^	44.46 ^ab^	162.67 ^ab^	19.50 ^a^	35.45 ^a^	38.83 ^ab^	64.17 ^ab^	217.33 ^b^	344.00 ^b^	207.30 ^b^	209.87 ^ab^

The studied species are abbreviated as follow: C.a.: *Cyperus alopecuroides*; E.s.: *Echinochloa stagnina*; P.a.: *Phragmites australis*; R.s.: *Ranunculus sceleratus*; T.d.: *Typha domingensis*; E.c.: *Eichhornia crassipes* and L.s.: *Ludwigia stolonifera*. Different letters indicate significant differences among species as well as metals at *p* ≤ 0.05 according to the Kruskal–Wallis test.

**Table 2:** Results of three-way analysis of variance assessing the effect of the drain (three drains), species (seven species), and tissues (aboveground and belowground) on the detected heavy metals.

Variable	Heavy Metal							
	Fe	Cu	Zn	Mn	Co	Cd	Ni	Pb
Drain (D)	239.25 ***	1.65 ^ns^	3.29 **	8.63 **	0.03 ^ns^	7.46 **	1.20 ^ns^	112.20 ***
Species (S)	20.18 **	30.15 **	45.60 **	12.10 ***	5.90 **	22.50 ***	7.50 ***	8.23 **
Tissue (T)	1.50 ^ns^	85.10 ***	10.90 ***	5.80 ***	1.45 ^ns^	8.45 ***	23.10 ^ns^	2.80 ***
D × S	5.40 ***	3.50 ***	5.30 ***	84.12 **	5.20 ***	22.40 ***	10.15 ***	11.00 **
D × T	7.50 ^ns^	0.85 ^ns^	10.50 ***	5.40 ***	1.50 ^ns^	3.20 **	0.75 ^ns^	9.12 **
S × T	5.30 ***	3.12 **	10.90 ***	2.50 ***	10.09 **	5.50 ***	7.30 ***	4.10 **
D × S × T	15.30 ***	3.50 **	4.20 **	8.10 ***	5.20 ***	3.80 **	9.10 **	5.50 ***

**: *p* ≤ 0.01, ***: *p* ≤ 0.001, and *ns*: not significant (*p* > 0.05) according to Kruskal-Wallis test.

**Table 3:** Bioaccumulation factor (BF) and translocation factor (TF) of heavy metals in the studied species. Highlighted values represent BF and TF mean values greater than 1.

Heavy Metal	Plant Species													
	C.a.		E.s.		P.a.		R.s.		T.d.		E.c.		L.s.	
	BF	TF	BF	TF	BF	TF	BF	TF	BF	TF	BF	TF	BF	TF
Fe	0.25 ^a^	0.89 ^a^	2.43 ^ab^	0.79 ^a^	3.83 ^b^	0.72 ^a^	0.32 ^a^	0.43 ^a^	1.52 ^ab^	0.65 ^a^	3.42 ^ab^	0.43 ^a^	0.26 ^a^	0.85 ^a^
Cu	0.07 ^a^	0.34 ^ab^	0.27 ^ab^	0.55 ^ab^	0.34 ^ab^	0.29 ^a^	0.28 ^ab^	0.35 ^ab^	0.24 ^ab^	0.60 ^b^	0.60 ^b^	0.53 ^ab^	0.15 ^ab^	0.35 ^ab^
Zn	0.16 ^a^	0.58 ^ab^	0.74 ^ab^	0.80 ^b^	0.56 ^ab^	0.48 ^ab^	0.37 ^ab^	0.15 ^a^	0.30 ^ab^	0.83 ^b^	1.72 ^b^	0.58 ^ab^	0.43 ^ab^	0.44 ^ab^
Mn	0.07 ^a^	0.53 ^ab^	2.29 ^ab^	0.84 ^b^	3.99 ^ab^	0.69 ^ab^	0.12 ^ab^	0.36 ^a^	2.28 ^ab^	0.64 ^ab^	4.37 ^b^	0.65 ^ab^	0.38 ^ab^	0.61 ^ab^
Co	0.62 ^a^	0.47 ^ab^	0.98 ^ab^	0.65 ^ab^	5.49 ^b^	0.17 ^a^	1.02 ^ab^	0.53 ^ab^	3.29 ^ab^	0.38 ^ab^	2.89 ^ab^	0.70 ^ab^	1.21 ^ab^	0.79 ^b^
Cd	0.40 ^ab^	0.55 ^ab^	1.05 ^ab^	0.53 ^ab^	5.78 ^b^	0.26 ^a^	0.22 ^a^	0.89 ^ab^	1.83 ^ab^	0.50 ^ab^	1.67 ^ab^	0.79 ^ab^	2.02 ^ab^	1.59 ^b^
Ni	0.37 ^a^	0.46 ^ab^	3.18 ^ab^	0.31 ^ab^	5.59 ^b^	0.19 ^a^	2.42 ^ab^	0.25 ^ab^	4.02 ^ab^	0.45 ^ab^	4.25 ^ab^	0.81 ^b^	3.52 ^ab^	0.73 ^ab^
Pb	0.65 ^a^	0.77 ^ab^	2.59 ^ab^	0.34 ^ab^	2.87 ^ab^	0.27 ^a^	0.62 ^a^	0.55 ^ab^	1.13 ^ab^	0.60 ^ab^	6.06 ^b^	0.63 ^ab^	3.69 ^ab^	0.99 ^b^

The studied species are abbreviated as follow: C.a.: *Cyperus alopecuroides*; E.s.: *Echinochloa stagnina*; P.a.: *Phragmites australis*; R.s.: *Ranunculus sceleratus*; T.d.: *Typha domingensis*; E.c.: *Eichhornia crassipes* and L.s.: *Ludwigia stolonifera*. Different letters display significant differences among species as well as heavy metals at *p* ≤ 0.05 according to the Kruskal–Wallis test.

## Data Availability

Not applicable.
